# Nanopipettes as Monitoring Probes for the Single Living Cell: State of the Art and Future Directions in Molecular Biology

**DOI:** 10.3390/cells7060055

**Published:** 2018-06-06

**Authors:** Gonca Bulbul, Gepoliano Chaves, Joseph Olivier, Rifat Emrah Ozel, Nader Pourmand

**Affiliations:** Department of Biomolecular Engineering, University of California, Santa Cruz, CA 95064, USA; gbulbul@ucsc.edu (G.B.); gchaves@ucsc.edu (G.C.); joolivie@ucsc.edu (J.O.); ozel.emrah@gmail.com (R.E.O.)

**Keywords:** nanopipette, single cell, nanobiopsy, nanogenomics, sensing

## Abstract

Examining the behavior of a single cell within its natural environment is valuable for understanding both the biological processes that control the function of cells and how injury or disease lead to pathological change of their function. Single-cell analysis can reveal information regarding the causes of genetic changes, and it can contribute to studies on the molecular basis of cell transformation and proliferation. By contrast, whole tissue biopsies can only yield information on a statistical average of several processes occurring in a population of different cells. Electrowetting within a nanopipette provides a nanobiopsy platform for the extraction of cellular material from single living cells. Additionally, functionalized nanopipette sensing probes can differentiate analytes based on their size, shape or charge density, making the technology uniquely suited to sensing changes in single-cell dynamics. In this review, we highlight the potential of nanopipette technology as a non-destructive analytical tool to monitor single living cells, with particular attention to integration into applications in molecular biology.

## 1. Introduction

Nanopipettes are of scientific interest due to their application potential in several arenas, from biomedical diagnostics to cellular biology. Nanopipettes are characterized by the submicron to nanoscale size of the pore opening at the tip, which serves as a suitable surface to fabricate functional tools for delivery to and/or aspiration from a single living cell, or for probing the cell’s contents. The hollow structure enables the dispensation of fluid from one region to the next, with their cavity acting as passage [1]. In view of the fact that many biologically significant molecules, such as DNA and proteins, are not able to spontaneously cross the cell membrane [2], the use of a non-destructive single cell manipulation platform such as nanopipettes to study single-cell dynamics is rapidly increasing. Other analysis techniques that require dissociation of tissue from its natural environment lead to the loss of spatial information on individual cells. Previous efforts at single cell manipulation include microinjection to introduce molecules into the cytoplasm of single cells [3]; microfluidic technologies [4,5], scanning probe and atomic force microscopy [6] to extract various biomolecules from the cell cytosol. Nanopipettes offer significant advantages over these techniques in that they target a specific single cell and the particular parts of the cell, including the nucleus, and the ability to inject the cargo precisely.

The fundamental understanding of the molecular biology of single living cells in heterogeneous cell populations is of the utmost importance in assessing changes in cellular functions in tissues. Whole tissue biopsies can provide information on many events that are occurring in different cells, but difficulties not always suitable for drawing conclusions regarding the progression of some diseases. For example, malignant tumors are heterogeneous in most cases and can include cells at different stages of transformation [7]. Because they provide a tool that both can inject molecules into a cell and also probe the presence of biomarker molecules, nanopipettes are useful in correlating the cellular mechanism of one disease with another, as was recently demonstrated for Huntington’s and intracellular glucose levels [8]. Thus, the use of multi-functional nanopipettes in single cell interrogation is beneficial in understanding the mechanism and pathways that link two related conditions, aiding in the development of drug therapies, and at the same time contributing to diagnostics for at-risk individuals. Tools such as nanopipettes, which are easy to adapt to several fields by modifying the nanopipette with different functionalities, can find application in many scientific disciplines [9,10,11,12,13].

Pipettes have been employed to transfer specified volumes of liquids in science and medicine for centuries [14]. The use of glass micropipette as an intracellular microelectrode was shown as early as 1902 [15]. Later, the increasing need for precise manipulation of small volumes in molecular biology resulted in the production of micropipettes with the ability to dispense volumes in the μL to mL range. Pipettes were used in the patch-clamp method in 1976 by Neher and Sakmann for detection of voltages and current from ion-channels [16]. Most recently, with the advances in electrophysiology and manufacturing at the nanoscale, nanopipettes emerged as useful tools for both in controlling and depositing small volumes, and in analytical sciences. Previous publications have summarized the production and characterization of different types of nanopipettes [17]. In this review, we focus on the different areas of application of nanopipettes in molecular biology, which include their use as: (1) surgical tools to inject or aspirate molecules from single living cells; (2) functional probes to monitor the presence of biologically relevant molecules in single cells.

## 2. Use of Nanopipettes as Surgical Tools

### 2.1. Nanoinjections by Single-Cell Surgery

Recently, information illuminating the behavior of single cells has received a great deal of attention [18,19]. To assess the response of a single cell, it is necessary to have an instrument capable of rapidly analyzing and manipulating individual cells in an automated way, while avoiding any damage that could affect these cells’ viability. Conventional methods of cell injection employ micropipettes [20] that deliver a large volume of substance that is incompatible with the size of typical cells. In other methods, such as atomic force microscopy (AFM), hollow cantilevers [21] were constructed, but these are also limited by lack of control of injection volumes. Electrochemical autosyringes that deliver the cargo by applying voltage across the liquid/liquid interface [2] and double-barrel glass nanopipettes capable of controlled deposition of biomolecules onto functionalized surfaces [22] showed potential for injections through cell membranes. Previous studies examined the injections of and aspiration from the cells based on microfluidic devices, light pulses, and photothermal nanoblades [23,24,25,26]. The main advantage of our nanopipette/nanosensor over these methods is the electrical system, which has a built-in feedback mechanism using homemade software that allows the user to find the cell and penetrate in an automated manner.

**Figure 1:**
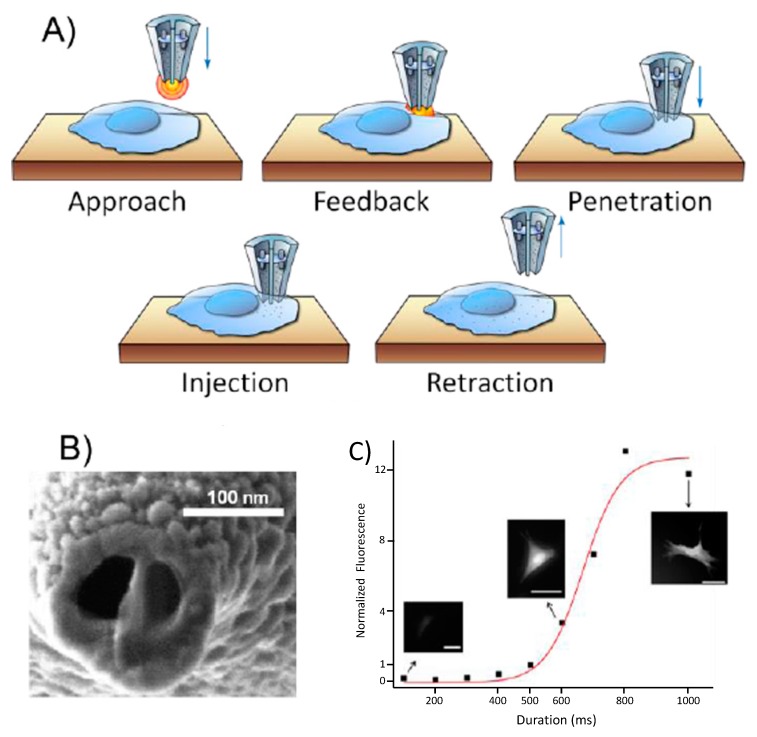
(**A**) Schematic representation of cell-surface detection by a double-barrel nanopipette; (**B**) SEM image shows the gold-sputtered double-barrel nanopipette; (**C**) Injection of carboxyfluorescein into human fibroblasts. The fluorescence intensity was normalized to that measured at 500 ms. Applied voltage: 10 V, scale bars 50 μm. The red curve is a sigmoidal fit to the experimental data points. (Reproduced from [27] with the permission of the Royal Society of Chemistry).

Our group has presented the development of a fully electrical system that makes it possible to inject a controlled amount of material (~50 fL) into a single cell [11]. We demonstrated the deployment of the system with injections of fluorescent dyes into adherent mammalian cells [27]. A Scanning Ion Conductance Microscope (SICM) was integrated in the platform so we could differentiate a single cell from the population of cells. The system can detect the target cell surface and enables the delivery of molecules into individual cells by employing voltage pulses. Unique advantages of our system include simultaneous cell surface detection and control over the volume of cargo delivery, which were not included in previous nanoscale injection systems [28,29]. A nanopipette is used in our cell injection system both for feedback-controlled nanopositioning and for delivery. The system is designed to work by following five steps: approach, feedback, penetration, injection, and retraction, as depicted in Figure 1. A detailed explanation of the feedback mechanism and the protocol can be found in Seger et al., 2012 [27]. The post-injection long-term cell viability was monitored by injecting carboxy-fluorescein succinimidyl ester into the cell and following the its morphology. Normal cell division was observed after 27 h of injection, with the daughter cells having normal cell morphology and migratory projections [27]. Future advances in the technology would enable a fully automated system for multiplex single-cell injections at the same time. Furthermore, interactions between different cell components, such as protein-RNA interactions, can be studied with this nanoinjection platform. RNA-binding proteins regulate the processes that RNAs are subjected to during and after transcription. Understanding protein-RNA interactions is crucial in illuminating their effect on the fate and function of RNA molecules. With our ongoing progress in monitoring the presence and concentration of proteins, nanopipettes can be deployed for real-time detection of complex protein interactions in the future. Further information on the transcriptome can be revealed by nanobiopsy and correlated with the behavior of the RNA-binding proteins [30].

#### Intracellular Tracking of Injected Components

Various molecules can be used to track a cell, such as genetically encoded fluorescent proteins [31], quantum dots [32], and fluorescent dyes. The ability to follow the process of the division of a single cell and how it transfers information to its daughter cells is key to advancing molecular biology and genomics. This ability would also be of benefit to developmental biology in understanding the conversion of a single cell into a full organism [33]. The ability to analyze the lineage history of cell populations would reveal information on developmental origin [34] and contribute to studies of genetically transformed diseases [35]. Additionally, predicting the behavior and function of a single cell in complex tissue over time could contribute to pharmaceutical development and personalized medicine [36] by providing targets upstream in pathogenesis. More efficient drug therapies, earlier intervention and recovery are sought to improve patient treatment and quality of life. Drug resistance during treatment is one of the major problems in many diseases, particularly in cancer [37,38,39,40]. Resistance to a drug can even result in the resistance of cells to other pharmaceuticals [41], decreasing the chances of successful treatment. Therefore, it is crucial to examine the population of cells where resistance is developing in order to understand the molecular basis of drug resistance and to improve treatment outcome. In order to overcome drug resistance, researchers must understand how genomic changes are transferred from one cancer cell to another, including the perpetuation of drug resistance. Identified alterations can reveal genetic signatures of the development of drug resistance, leading to earlier intervention, modulation of therapy, and improved treatment outcomes.

### 2.2. Single-Cell Nanobiopsy Platform

A single-cell nanobiopsy platform was developed for continuous sampling of intracellular content from individual cells and has been described in detail elsewhere [11]. Because it is possible to extract a minute volume of material with a nano-size tip, we can deploy our custom single-cell biopsy platform for the extraction of cytoplasm from multiple locations in the same cell. The ability to map the subcellular distribution of different biomolecules opens up new avenues of study; it is now possible to obtain information on cellular circuitry, neuronal growth, and network formation among cells, contributing to proteomics, genomics and diagnostics.

#### 2.2.1. Single Cell Immunoassay

Aside from carbohydrates and nucleic acids, proteins are one of the most common macromolecules in cells. Among these molecules, proteins are the most diverse molecules, playing a variety of biological roles: communication of information within and among cells, protection of cells against infection, catalysts for chemical reactions, and as structural components, to name but a few [42]. Therefore, there is great interest in quantifying, identifying and isolating proteins, in order to understand the plethora of unknown mechanisms in which they are involved. Conventionally, the methods of Lowry and Bradford were employed to quantify total protein content [43,44]. However, these methods do not permit the identification of specific proteins involved in the processes of living cells. Subsequently, antibodies were utilized to identify specific proteins [45], and Southern blot, Northern blot and Western blot analyses were developed to detect DNA, RNA, and proteins, respectively. The Western Blot was then adapted to detect single-cell proteins differentiated by molecular weight; this enabled the interrogation of more than 1000 cells in less than 4 h and multiplexed measurements of up to 11 proteins [46]. Single-cell Western blot analysis, however, relies on separating the single-cell protein lysate using a polyacrylamide gel coating on a glass microscope slide, which can be destructive. Flow cytometry, microfluidics technologies, and surface methods such as ELISPOT were also studied as single-cell functional proteomics tools and have been extensively reviewed elsewhere [47]. The use of functionalized nanopipettes as a platform for label-free identification of biomolecules such as proteins has been strongly recommended. Also, protein-based recognition elements, such as antibodies and enzymes, can be functionalized in the sensing zone and further used for sensing of various molecules [48] (a summary of sensing applications is given in Section 2 below). Functionalized nanopipettes can then be inserted into the single cell and used to monitor proteins in that cell. An antibody-labeled nanopipette shows excellent potential for the longitudinal interrogation of single cells. Implementation of this technology is on the cutting edge of advances in developing methods to combat human diseases. In addition to the proteomic approach, incorporating aspiration and sequencing of molecules from the nanopipette biopsy could identify significant disease-resistant variant genes. Therefore, the nanopipette can serve as a platform for integrated analyses in genomics, transcriptomics, proteomics and metabolomics.

#### 2.2.2. Genomics

The Human Genome Project pioneered research to identify the genetic entities behind conditions such as genetic disorders and drug resistance [49]. This research has become key in the process of drug discovery. However, drug discovery and diagnostics continue to present significant challenges. One difficulty lies in the heterogeneity of cells in complex tissues. The need to overcome this difficulty motivated the development of useful tools for single-cell genomics and transcriptomics. These methods allow the examination of individual cells, circumventing the need to interpret pooled genetic information in population-based experiments, which will mask the earliest stages of change. Drug resistance, for example, can originate with mutations in an individual cell, which can then take over an entire cell population [7]. To overcome research limits imposed by averaging out subpopulations in heterogeneous tissue, single-cell interrogation has advanced in recent years. Single-cell investigations that are destructive do not represent appropriate tools, because it is necessary to scrutinize genetic variation arising from the same cell over time. Some biological processes require monitoring of the same cell at multiple time points to understand the complete process. Fluorescence time-lapse microscopy was previously used to analyze gene circuit dynamics and heterogeneous cell behavior [50]. As an example, this technology was applied in embryonic stem cells to reveal the dynamics of the expression of pluripotency factor Nanog. Microscope-based detection of expression assessed different expression levels of the Nanog protein, demonstrating the interchangeable levels of Nanog-high and Nanog-low cells [51]. We deployed our nanopipette technology as a tool to aspirate the genomic information from single living cells and sequence the code with no destructive effect on the cell membrane [11]. The nanopipette platform enables non-destructive intermittent interrogation or continuous longitudinal interrogation of single cells, and has the advantages of a non-destructive, label-free, single-cell monitoring system. As well, the nanopipette method allows one to assess gene expression in subcellular compartments and organelles such as the cytoplasm, nucleus, and mitochondria.

#### 2.2.3. Single Cell Aspiration

**Figure 2:**
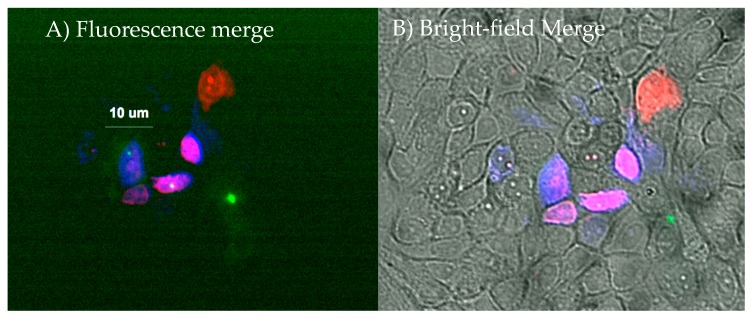
(**A**) Fluorescence; (**B**) Bright-field merges show injections of green fluorescent protein (GFP), rhodamine, and mitotracker orange into the cells. GFP: green channel; mitotracker orange: blue channel; rhodamine: red channel. Cells stained purple are a mix of blue (mitotracker) and red (rhodamine) channels. One cell at center can be seen with GFP, mitotracker and rhodamine fluorescence, indicating three independent nanopipette interrogations. GFP was the first component to be injected into the cell, however it did not diffuse well into the cell, probably due to protein viscosity. After GFP, mitochondria-staining dye mitotracker orange was introduced. Rhodamine was injected as the third component into the group of cells. (Pourmand Lab, Personal Communication, 2018).

Extraction of molecules from a single cell by means of the nanobiopsy platform relies on electrowetting within a nanopipette. In brief, when an organic solution fills a nanopipette and the device is inserted into an aqueous solution, a liquid-liquid interface is created at the tip. Once voltage is applied between these two solutions, a force is produced at the interface that causes the solution to enter into or leave from the nanopipette [2,52]. Under this condition, when a negative potential is applied, the solution moves toward the lumen of the nanopipette, and when a positive potential is applied, the solution moves to the outside of the nanopipette. In these interrogations, the amount of aspirated material from the cell compartment was estimated to be around 50 fL, or approximately 1% of the volume of a cell [11]. As mentioned above, we integrated the nanopipette platform into a scanning ion conductance microscope (SICM) system that automatically positions the nanopipette above the cell of interest [27]. While in aqueous solution, the nanopipette is biased with a positive voltage to prevent the solution from flowing towards the lumen of the nanopipette. This voltage generates an ion current between the liquid-liquid interface, which can be used as the input of a feedback loop integrated with custom-built software. The software controls the movement of the nanopipette, continuing to approach the cell until a drop in the ionic current is detected, indicating the tip is at close proximity to the surface of the cell [27]. When a reduction of the electric current is detected, the software stops movement in the direction of the cell and lowers the nanopipette at high speed (100 μm/s). This movement inserts the nanopipette into the cell membrane. The voltage applied to the nanopipette is then switched to 500 mV for 5 s, causing aspiration of cell cytoplasm into the nanopipette. Subsequently, a switch to 100 mV stops the influx, but does not induce the efflux of the aspirated content [11]. Nanopipettes fabricated from multiple-barreled capillaries allow the simultaneous injection of dye as molecules of biological interest are aspirated from the cell. Because of the small size of the device (approx. 50 nm), injury to cells from the nanopipette is minimal. Sequential delivery of multiple dyes has demonstrated the ability of the nanopipette platform to interrogate the single cell numerous times without fatally damaging the cell. Figure 2 shows the injection of multiple dyes into a single cell. Seger and collaborators demonstrated the ability of cells to survive for 27 h after the exposure [27]. These injections suggest the potential application of the nanopipette platform in multiple interrogations of the single cell, without lethal damage, which can be critical for the development of single-cell drug resistance studies. Another study showed the use of nanopipettes to detect genes that were not previously described in the body of a neuron [53] by finding the compartmentalization of mRNA molecules in different parts of neurons. For the mRNA molecule of interest to be interrogated, it must first be sequenced.

#### 2.2.4. Nanogenomics

The nanopipette can also be employed to aspirate cell contents from the same single cell multiple times during its lifetime to study molecular dynamics. This platform was previously validated to isolate molecules such as RNA for cDNA synthesis and qPCR, and our group became one of only a few to have performed next-generation sequencing (NGS) from the species extracted with nanopipettes. Nashimoto’s research group has shown device automation in the ZYX axis for isolation of mRNA molecules [54]. Guillaume-Gentil has demonstrated the identification of metabolites and enzymes using atomic force microscopy and also validated mRNA aspiration using qPCR [55]. However, analytical techniques such as NMR and MS spectrometry for the detection of single-cell molecules are still limited. In 2007, Luo and Li reported on the identification of 12C/13C-dansyl labeled metabolites by means of MALDI-MS in a minimum of 100 cells [56]. The group was able to detect subpopulations of heterogeneous tissue, but technical limitations of the method did not allow single-cell resolution. Guillaume-Gentil also reported the utilization of atomic force for aspiration and detection of mRNA molecules [57]. Cao and collaborators demonstrated longitudinal interrogation of single cells, sampling GFP and RFP transcripts from cells [58]. These techniques, on the one hand, relied on the observation of aspiration by fluorescence or qPCR amplification. Genes of interest, on the other hand, are not always tagged with fluorescent protein to identify protein localization. Also, not all RNA molecules involved in genetic mechanisms are expressed as proteins. However, it is not rare that all the genes of a cell must be interrogated. To successfully identify the highest possible number of genes involved in drug resistance, interrogation of cells can only be accomplished using next-generation DNA sequencing platforms.

To show that nanopipettes did not affect in the function of cells upon piercing the cell membrane, human BJ fibroblasts were treated with Ca^2+^ agent Fluo4 AM, and fluorescent microscopy was used to show the localization of Ca^2+^ ions before, during and after nanopipette biopsy [11]. Optic microscopy images showed that the procedure was minimally invasive, generating only a small change of Ca^2+^ during nanobiopsy. The cell recovered a few seconds after the process, reaching Ca^2+^ concentrations that matched pre-aspiration levels. By contrast, Actis et al. demonstrated that micropipette aspiration caused dramatic changes in the concentration of Ca^2+^ ions in the cell [11]. The low interference of nanopipettes results from the minimal interaction of the nanopipette with the surface membrane of the cell, in contrast with the highest surface of communication and damage demonstrated by micropipettes.

It is important to note that nanopipette aspiration is based on a voltage-controlled influx of material and not adsorption of molecules to the walls of nanopipette. PCR amplification of DNA templates was not observed if negative voltage was not applied to the nanopipette during single-cell interrogation and when aspiration was performed in the bulk solution. This is the critical element that differentiates the nanobiopsy technology from AFM-based platforms. Both Wickramasinghe’s and Osada’s groups used AFM probes to extract RNA from cells in culture, either based on physisorption or hybridization of complementary RNA immobilized onto the probe [59,60]. We can foresee that the use of nanopipettes to aspirate limited copies of mitochondrial DNA from a living cell might provide the basis for less invasive and more accurate monitoring of disease progression. The potential of nanobiopsy is also such that the foundation can be established for the development of new classes of drugs to attenuate diseases as diverse as Parkinson’s and Alzheimer’s Disease. The nanopipette can be used as a platform for cancer research and clinical management, elucidating the role of heterogeneity in primary tumor tissues and systemically identifying critical parameters in disease progression and potential metastatic states [61,62]. By combining the nanopipette platform with downstream sequencing implementation, gene expression inside single cells can be longitudinally investigated, and the effect of drug mechanisms on mutation-selection can be better examined.

**Figure 3:**
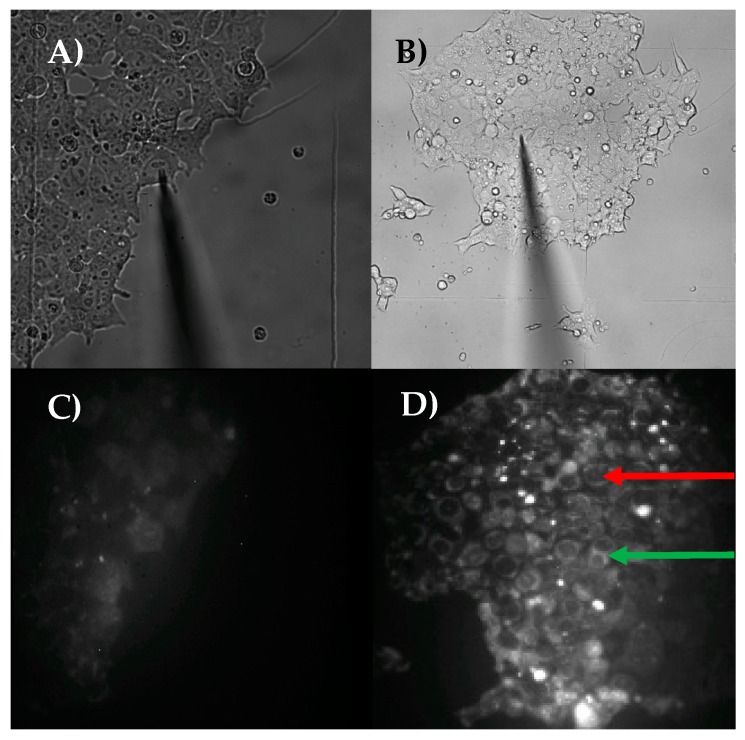
Aspiration of nuclear content by Nanopipette. (**A**) Nanopipette is placed on top of MCF-7 cell; (**B**) Nanopipette is placed on top of a different MCF-7 cell; (**C**) Fluorescence corresponding to mitotracker orange staining of cells depicted in (**A**); (**D**) Fluorescence corresponding to mitotracker orange staining of cells depicted in (**B**). Nuclear region is visualized by pattern of staining with the mitotracker dye. In (**D**) red arrow points dark compartment, corresponding to one nucleus. Green arrow shows one cytoplasmic region. Nanopipette was inserted into the nucleus, as seen in (**B**). Nuclear content was aspirated and transferred to the cDNA synthesis master mix, followed by sequencing using the Illumina Miseq. (Pourmand Lab, Personal Communication, 2018).

The nanopipette platform also allows subcellular interrogation. By using different dyes in the cellular nucleus or by staining the cytoplasm, enabling the isolation of the nucleus, it is possible to target the two compartments differentially. The following pictures in Figure 3 show cells stained with mitochondria dye (mitotracker orange). The chromosomal region can be distinguished from the cytoplasmic by observing the white granulocytes that correspond to the interaction of mitochondrial proteins with the dye. The nucleus is depicted as circular black orifices without mitochondria. The nanopipette was inserted into the dark orifice, corresponding to the cellular nucleus.

**Figure 4:**
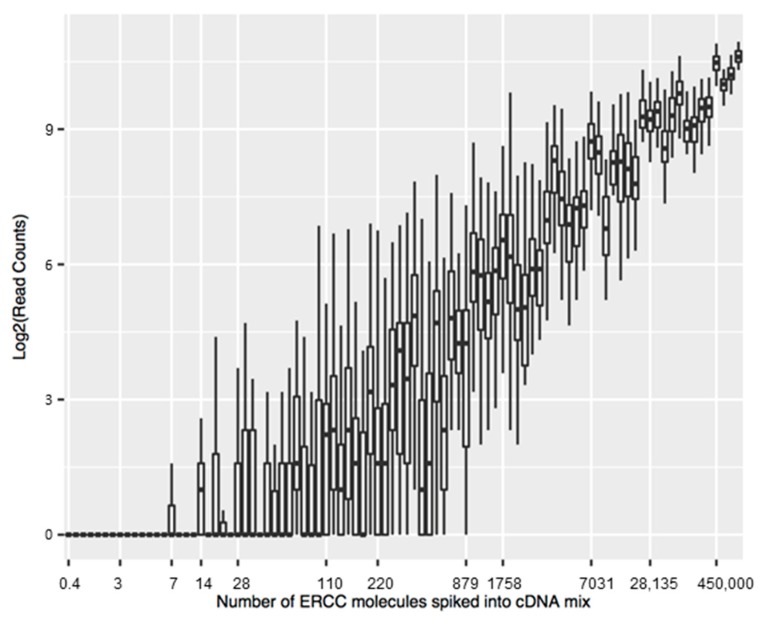
Limit of detection of ERCC. Content from the nanobiopsy of the nucleus was transferred to the cDNA mix (containing 0.5 µL of ERCC mixture at a 1:10,000 dilution) to reverse transcribe the RNAs followed by DNA sequencing. The sequencing reads were mapped to the ERCC reference pseudo-genome. The number of transcripts were counted using the HTSeq package and plotted as a function of the number of the ERCC transcripts (ERCC concentration × volume × dilution factor). The estimated intersect of the ERCC curve with the X axis was between 7 and 220, which represents at least one detected ERCC transcript. The threshold for detected transcripts was chosen to be 10 for subsequent analysis. (Pourmand Lab, Personal Communication, 2018).

To control the sequencing process of nanopipette aspiration downstream, we implemented the addition of External RNA Controls Consortium (ERCC) spike-in controls with samples collected from the cells. ERCC controls are a set of RNA standards for use in microarray, qPCR and sequencing applications [63]. These molecules are artificial poly-adenylated RNA, used in library preparation protocols before cDNA synthesis. We detected increased variability as the number of detected reads decreased. However, the nanopipette biopsy was able to identify reads mapping to the human genome [53]. Therefore, coupling the nanopipette platform with the sequencing of mRNA molecules showed the ability of the nanopipette platform to successfully identify low-abundant molecules in the context of gene expression, a capability essential for single-cell interrogation. ERCC spike-ins were used to show the ability of nanopipettes to isolate cellular RNA molecules for sequencing. Reads were used that mapped to at least one spot in the human genome, as described by Actis et al. [11] and Toth et al. [53]. Figure 4 shows the number of reads (counts) mapped to the ERCC reference as a function of ERCC concentration.

**Figure 5:**
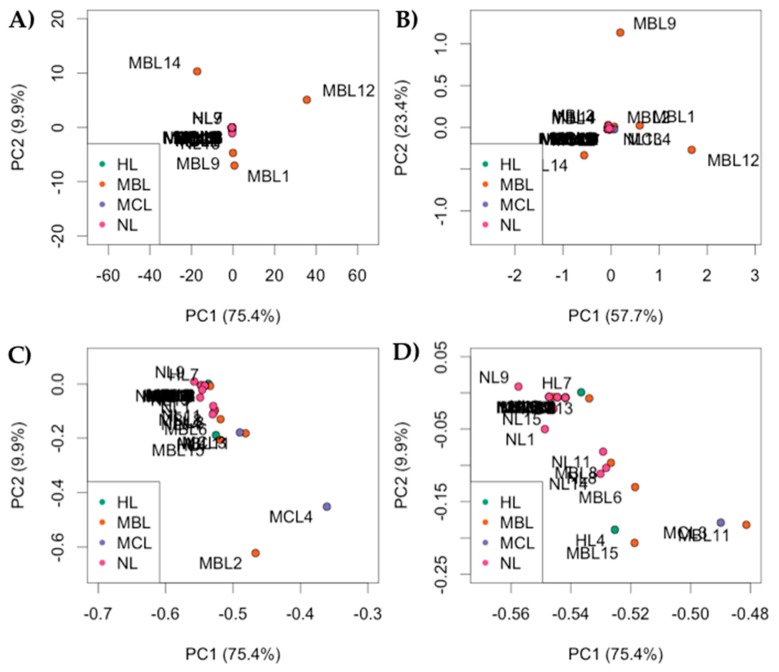
Principal Component Analysis of gene expression in the nuclear nanobiopsy samples. (**A**) Raw data input to DESeq2; (**B**) DESeq2 run with log-normalized reads; (**C**) Resolution of clustering after removal of the MBL1, MBL9, MBL12 and MBL12 libraries as outliers; (**D**) Resolution of clustering excluding sequencing libraries MBL2 and MBL4. (Pourmand Lab, Personal Communication, 2018).

After separation of the ERCC counts from reads proceeding from cellular content, reads mapped to the human reference genome were plotted as Principal Component Analysis (PCA) results of cellular expression, showing the clustering pattern of the nuclear aspirations of single cells. The PCA of gene expression in the nuclear nanobiopsy samples, using both non-processed and pre-processed gene counts, are shown in Figure 5. The sequencing reads were aligned against the human reference genome using the STAR aligner, and the HTSeq package was used to count the number of mapped reads. Using the limit of detection (10 reads per detected transcript), reads were input to DESeq2 (HL = HeLa transcriptome library; MBL = MDA-MB-231 transcriptome library; NL = iCell neuron library; MCL = MCF-7 transcriptome library). Figure 5A shows the PCA of gene expression in the nanobiopsy samples. Libraries MBL1, MBL9, MBL12, and MBL14 were considered outliers and removed from downstream analysis. The Figure 5B graphs are plotted from PCA runs with reads log-transformation, with the aim of mitigating the variation effect of highly expressed genes or any biases possibly introduced during the cell nanobiopsy procedure, library preparation or sequencing run. In Figure 5C we focused on the areas from Figure 5A that have more clustering structure for better visualization. Furthermore, Figure 5D was plotted to give a closer look to the clustered areas from Figure 5C. Figure 5C,D illustrate the expression profile of the four cell types are different from one another.

**Figure 6:**
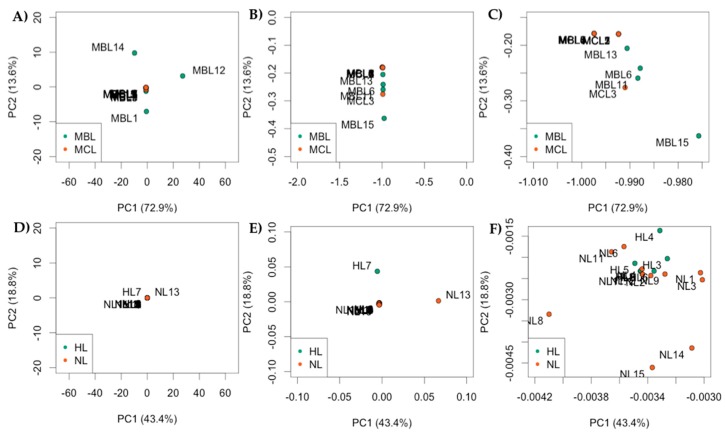
Principal Component Analysis of gene expression comparing two cell types at a time. (**A**–**C**) comparison of MDA-MB-231 and MCF-7 libraries cluster separately by cell type, seen as a trend in which same-cell type libraries cluster closer to each other; (**D**–**F**) comparison of HeLa vs. iCell Neurons cells. Libraries cluster separately by cell type. (Pourmand Lab, Personal Communication, 2018)

It was not clear to what extent the MDA-MB-231 cells and MCF-7 cells were distinguishable using PCA in Figure 5. Therefore, we plotted the MDA-MB-231 cells and MCF-7 separately in (Figure 6A–C). Figure 6B represents focused areas of Figure 6A, Figure 6C represents focused areas of Figure 6B, for more clustering structure. Figure 6E represents focused areas of Figure 6D for more clustering structure. These results suggest that, although the number of detected reads is small per sequenced libraries, nanopipette technology detects the similarities of same-cell type. Figure 6D–F support the conclusion of MDA-MB-231 vs. MCF-7 comparison. Figure 6E represents focused areas of Figure 6D, Figure 6F represents focused areas of Figure 6E, with more clustering structure.

To determine the identity of more abundant genes in the MDA-MB-231 and MCF-7 cells, we extracted RefSeq IDs with more than 200 reads in at least one of the 39 sequenced libraries from the dataset, and checked the presence of the genes in both MDA-MB-231 and MCF-7 cells. Table 1 represents the ability of nanobiopsy to resolve the identity of a cell type by detecting highly abundant transcripts associated with ubiquitous biological processes, as an example, genes associated with glucose metabolism (UGP2, ENO1), ribosomal protein synthesis (RPLP0, EEF1A1, NPM1), protein folding (HSP90AA1), protein degradation (POMP), DNA binding (H3F3B), and drug resistance by cancer cells (AXL) [64,65,66,67,68,69]. More specifically, genes ENO1, H3F3B and HSP90AA1 are important cancer drivers in human cells.

## 3. Monitoring Intracellular Components by Using Nanopipettes: Sensing

The identification and quantification of molecules in the single cell play a crucial role in diagnostics and fundamental molecular biology. The ability to dynamically monitor the presence and amount of any molecule and/or biomarkers in the single cell aids in understanding the relationship of these molecules to several diseases, and contributes to drug discovery research. The sensing region or the nanopipette tip surface responds to changes in the ionic current flowing through the pore, which can be brought about by electrostatic, biotin-streptavidin, or antibody-antigen interactions. Specific antigen-antibody interaction changed the current amplitude and showed reasonable promise for future applications in biomolecular diagnosis. The successful implementation of nanopipette technology in biosensing enabled the identification of a variety of molecules from glucose to proteins. In the following section, we review the commonly used immobilization techniques, techniques to generate signal, and recognition probes on the nanopipette. Specific examples from the literature are given subsequently.

### 3.1. Layer-by-Layer (LbL) Immobilization of Recognition Elements

The idea of running voltage through the nanopore and using the resultant current as feedback originated in 2002 [70]. Beginning in 2004, numerous groups began using nanopipettes as a transport system for metals and small molecules [22,71,72]. In 2006, the idea of attaching polymers to the nanopipette in order to increase feedback responses came to fruition, when was shown that the surface charge could be manipulated by coating the pore with Poly-L-Lysine (PLL). The attachment of PLL was confirmed by the current rectification observed when running voltage through the tip of a coated nanopipette into a solution containing 25 mM KCl [73]. This feedback mechanism, which gave scientists the ability to confirm that a polymer was attached, allowed for further functionalization of nanopipettes in the form of layer-by-layer (LbL) assemblies. In 2009, Umehara et al. posited that the attachment of specific probes to the nanopipette pore could lead to label-free, quantitative sensing of small molecules, proteins, and/or antigens [48]. That paper was the first to detail an approach for LbL assembly that allowed the detection of specific proteins in solution using antibodies. First, PLL was coated and baked onto the bare surface of the nanopipette [74]. 1-ethyl-3-(3-dimethylaminopropyl) carbodiimide hydrochloride (EDC) and *N*-hydroxysulfosuccinimide (NHS) were then deposited to create a link between PLL and the subsequent layer [75]. Protein A/G was then conjugated to the NHS/EDC linker [76]. Finally, IgG was immobilized to protein A/G on the tip of the nanopipette [77]. During the same year, this type of LbL assembly was patented [78].

### 3.2. Electrochemical Techniques Used for Analysis

Nanopipettes have not always been functionalized into biosensors as they are today. Beginning in 2002, the Korchev and Klenerman labs began using nanopipettes as a new SICM probe for cellular structures and substructures [70,72,79,80]. In this setup, there is a reference electrode (RE) inside the nanopipette and a working electrode (WE) in the solution. Voltage is applied between the two electrodes, and the resultant current is used to gauge how far away the nanopipette is from the structure [68]. The nanopipette is attached to a piezo stage, which controls its xyz movement. Current is kept constant as the nanopipette is moved along the xy plane by adjusting its z position. By using this current feedback system, researchers are able to provide high-resolution imaging. Once a topological map has been drawn, the computer-controlled piezo stage can be used to precisely position the nanopipette over a feature of interest, such as an ion channel. Then, the current feedback system is switched off, allowing the nanopipette to be lowered to the surface of the channel. At that point, suction is applied, forming a giga-seal, which allows noise-reduced patch-clamp recordings to be made [81]. In addition to high-resolution imaging and patch-clamp recording, nanopipettes have been explored as vectors for molecule delivery.

At approximately this time, research began using nanopipettes for SICM; the labs cited above as well as others began to use nanopipettes to capture and transport DNA, metal ions, and other molecules [71,72,82,83,84]. In one example, carbon nanotubes were filled with iron; a current was run through the tube, and iron flowed out of the tube and was deposited on a surface [71]. Elsewhere, the same schematic was used as described above, where a reference electrode (RE) is placed inside the nanopipette while the working electrode (WE) is in a buffer solution [72,82,83,84]. In these cases, a potential waveform is applied between the electrodes that influences the electroosmotic flow (EOF), electrophoretic flow, and dielectrophoresis of DNA, metal ions, or proteins in solution. Klenerman’s group has reported variations observed when testing conditions are kept constant [82], suggesting that the electrical field and gradient inside the nanopipette is extremely sensitive to the geometry of the nanopipette.

In 2004, the Stanford Genome Technology Center began to use a nanopipette as an electrochemical biosensor [10]. Unlike previous works, the WE was placed inside the nanopipette, and the RE was placed in the bath solution. When voltage was applied between the two electrodes and the resultant current was measured, the Stanford group was able to observe translocation of DNA labelled with gold nanoparticles (DNA-AuNPs) by observing short bursts of current reduction when the DNA-AuNPs translocated into or out of the nanopipette. The same group began exploring the behaviors of functionalized nanopipettes. These experiments, which largely used the same electrochemical techniques, focused on current rectification of the nanopipette system at various applied voltages [73]. It was observed that coating nanopipettes with PLL could modulate current at particular applied voltages and could amplify signals produced under certain conditions. This led to the development of a layer-by-layer assembly on the inner pore of the nanopipette [48,78], with antibodies attached to its outermost layer, making it specific to a particular antigen. By applying a constant voltage, the researchers were able to see changes in current when the antigen was added to the solution. Variations of this assay exist in several forms. The Long lab, for example, has observed the ability to differentiate alpha-fetal protein (AFP) from AFP bound to its conjugate antibody (AFP-anti-AFP) [85]. In their experiments, they measured translocation events by observing the change in current as the protein or the antibody-bound protein transverses the nanopipette’s pore. It was shown that when the antibody binds to AFP the translocation is longer and more complete, causing a larger reduction in current. Today, the basic schematic for a nanopipette is to have two electrodes, one inside the nanopipette and one in the surrounding solution. A waveform is then applied through the electrodes and the resultant current is observed. The current can be modulated and changed based upon how the pipette has been functionalized or on the target for observation in the solution.

### 3.3. Recognition Element Selection for Immobilization on Nanopipettes

The recognition element is one of the major factors affecting nanosensor performance. The specificity of a nanopipette-based biosensor is restricted by the molecule deployed as the recognition element. Receptors, enzymes, antibodies, and nucleic acids can be employed in the sensor design to recognize the target of interest [86]. Initially, affinity reagents, such as monoclonal antibodies and enzymes obtained from living systems, were deployed in sensor construction. However, numerous concerns with monoclonal antibodies, such as reproducibility of the clone, high production costs, stability, and cross-reactivity, led to the development of oligonucleotide-based molecules for recognition [87]. Nucleic acid aptamers are attractive alternatives to protein-based recognition probes with lower cost, longer shelf life, and less batch-to-batch variation derived from the chemical production process [88]. Like antibody detection, aptameric detection takes advantage of specific binding between its conjugate and itself, which causes a change in current rectification that occurs as the pore of the nanopipette is blocked, and/or a change in the surface charge. The selection criteria for the recognition molecule of nanopipette-based sensing largely depend on the application area and the analyte. Using antibodies on the outermost layer can provide specific sensors; other molecules, such as aptamers [89] or enzymes [90], can readily be used as other recognition molecules for detection. Enzymes can also be used in a secondary detection method. For example, glucose oxidase was attached to the outermost layer of a nanopipette pore, causing the oxidation of β-d-glucose to d-gluconic acid. This led to a drop in pH, which caused a measurable change in current rectification [90].

### 3.4. Specific Examples from the Literature

#### 3.4.1. Glucose

**Figure 7:**
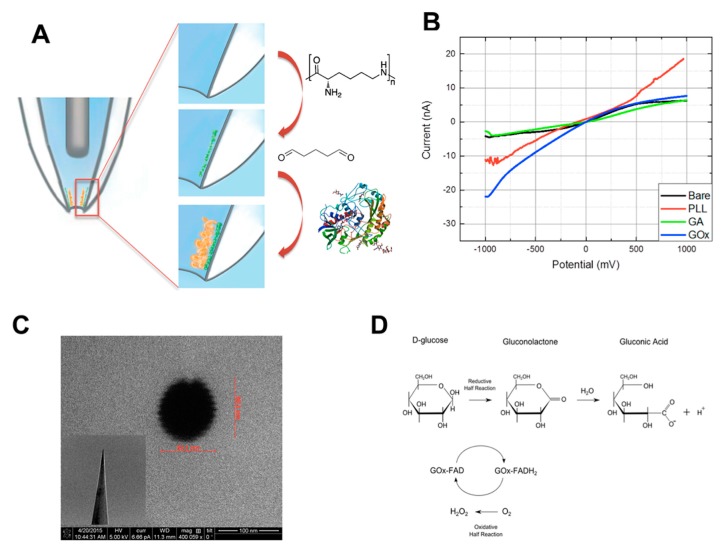
(**A**) Representative schematic showing the steps of glucose oxidase immobilization to the surface of the nanopipette tip. First, PLL is coated on the surface. Then, gluteraldehyde treatment occurs to cross-link the glucose oxidase to the PLL-coated surface; (**B**) After each step of immobilization, the changes were characterized electrochemically. 10 mM PBS (pH 7) was used as the supporting electrode; (**C**) Nanopipette tip imaged by SEM. Tip geometry is displayed in the inset; (**D**) Enzymatic process for conversion of glucose into hydrogen peroxide and gluconic acid. (Reprinted with the permission from [90]. Copyright (2018) American Chemical Society).

The differences between glucose levels of individual cells may be indicative of diseases such as cancer [91]. These changes can further assist in the identification of abnormal cells. After identification, these cells can be labeled and followed over the course of treatment. For example, increases in glucose levels were observed in the metastatic breast cancer lines MDA-MB-231 and MCF7 compared to nonmalignant cells [90]. This increase in glucose consumption contributed to the tumor cells’ rapid growth and proliferation, which requires increased metabolic activity. It was also speculated that altered glucose metabolism can result in metastasis and resistance to chemotherapeutic drugs [92]. Therefore, it is essential to monitor the metabolic activity of the single cell, not only for identification, but for the ability to study the transformation of single cancer cells in the heterogeneous cell population. We have used the nanopipette as a platform to immobilize glucose oxidase (GO) for real-time intracellular glucose detection and have monitored the changes in impedance [90] (Figure 7). A direct relation between impedance change and glucose concentration was observed and used to create a calibration curve. Notably, the surface chemistry developed for GO can further be employed for the attachment of various enzymes and applied to detect different substrates in the cells.

#### 3.4.2. pH and Reactive Oxygen Species

**Figure 8:**
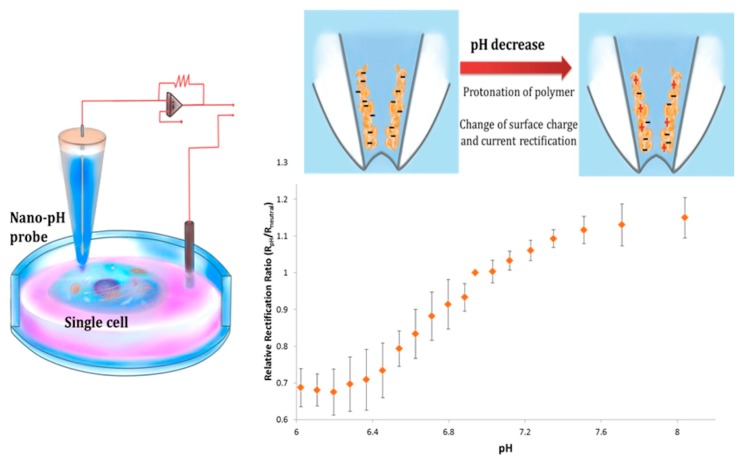
Schematic representation of electrochemical configuration and pH monitoring in a single cell with a chitosan-modified nanopipette. (Reproduced from [96] with the permission of the Royal Society of Chemistry).

Cytoplasmic ions and molecules can be used in the prediction of cell heterogeneity. Previously, it was found that the accumulation of metal ions [93] can be indicator of cancerous cells within a population. Additionally, changes in the levels of reactive oxygen species (ROS) and reactive nitrogen species (RNS) [94] were found to be indicators of cancerous cells within a population. Also, these cells were anticipated to be acidic [95] due to the high metabolic rate of cancer cells. In our efforts to investigate single-cell heterogeneity and build a relationship between pH and a variety of diseases, we developed a nanosized pH probe to measure pH at the single-cell level [96] (Figure 8). The development of this probe was accomplished by physisorption of chitosan onto hydroxylated quartz nanopipettes with a pore size of approximately 100 nm. The nanoprobe was capable of sensing pH in a range of 2.6 to 10.7, with a sensitivity of 0.09 units. The successful application of the probe in a single cell was performed on both non-cancerous and cancerous cell lines. Direct monitoring of intracellular pH was performed in human fibroblasts, HeLa, MDA-MB-231, and MCF-7 cells, with pH response to pharmaceutical manipulations.

Because changes in ROS levels in cancerous cells had been observed previously, the development of ROS single-cell monitoring systems will benefit studies of tumor progression [97]. Previous methods for intracellular ROS monitoring had significant limitations [98], such as unspecific generation of signal on the electrode surface deriving from the complex intracellular media. It is possible to overcome this challenge by taking advantage of the nanopipette platform, since it does not involve direct placement of the electrodes in the sample solution. Recently, we have functionalized nanopipettes to detect ROS levels and have successfully identified and quantified these levels in human cells (manuscript submitted).

#### 3.4.3. DNA

Single DNA molecules labeled with nanoparticles were detectible using glass nanopipettes by monitoring the ionic current blockade events caused by translocation of these molecules [10]. This technique not only provided a detection platform for single DNA molecules, but also yielded deeper insights and understanding of stochastic interactions of several molecules within their environment. 24-base single-stranded thiol-modified DNA labeled with 10 nm gold nanoparticles was sufficient to cause ionic current blockage events. After this approach was developed for the nanopipette DNA detection technique, monitoring of the folding state of double-stranded DNA in nanocapillaries [99] was demonstrated. Further research integrated the nanopipette with a microfluidic device and made it possible to discriminate between DNA molecules of varying lengths as they moved through the microfluidic channel [100].

#### 3.4.4. Metal Ions

**Figure 9:**
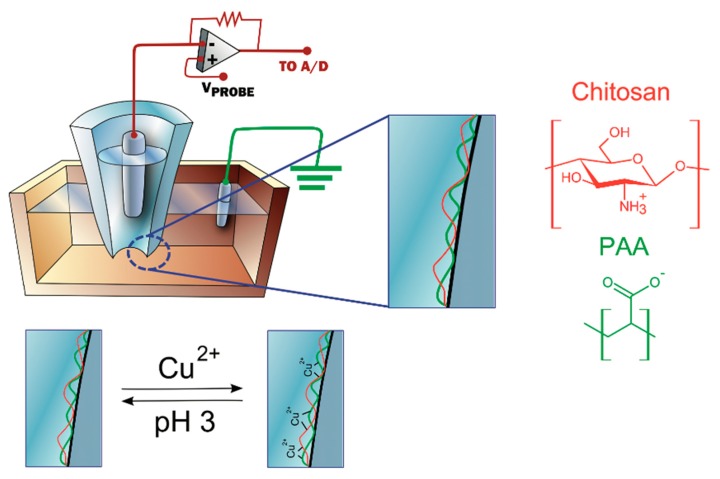
Schematics showing the electrochemical configuration (top left) and the reversible binding of copper ions on the chitosan/PAA-modified nanopipette. (Reprinted with permission from [101]. Copyright (2018) American Chemical Society).

Functionalization of a solid-state nanopore with chitosan and polyacrylic acid (PAA) made it possible to detect metal ions based on reversible binding of metal ions to polyelectrolytes [101] (Figure 9). The sensor was able to monitor the real-time signal of Cu^2+^ presence in a concentration-dependent manner. The ability to temporally direct the binding of molecules to a target contributes not only to the fabrication of sensing devices, but also to studies on the thermodynamics and kinetic properties of analyte-receptor interaction. The immobilization of calmodulin, a calcium-binding protein, enhanced the calcium ion response of the nanopipette and made possible the development of a sensor that presents selective and reversible binding of calcium at neutral pH [102]. In another report, quartz nanopipettes modified with an imidazole-terminated silane has been shown to produce a response upon cobalt binding. Adsorbed Co^2+^ was successfully released from the nanopipette surface by cycling the nanopipettes between solutions with different pH levels [103]. 

## 4. Conclusions and Future Perspectives

In the last decade, much effort has been concentrated on applications of nanopipettes as single-cell surgical tools both for injection and for aspiration of various materials. The combination of nanopipettes as surgical tools and selective sensing tools enabled detection of biologically relevant compounds in single living cells and in well-defined regions of the cell compartment. The advances in nanopipette technology have also benefited various areas of molecular biology research, including but not limited to proteomics and genomics. In the future, the technology presented here can be applied to automated cellular collection systems, which could allow the researcher to perform several tasks at the same time. As mentioned above, when a pipette is used as a biosensor the shape and the diameter of the tip is of greater interest than the overall volume. Therefore, there is significant interest in producing nanopipettes with precisely identical geometrical parameters, such as size and shape of the tip. Post-processing of nanoparticle production can help fine-tune these parameters by applying different approaches. Development of precise tip parameters is of scientific interest not only for sensing applications but would also be of interest to researchers dealing with dielectric etching. We believe that with increased attention to nanopores, nanogating, and ion channels, nanopipettes will find even broader application in a variety of fields, from electrophysiological to medical research, and will become a fundamental tool for single-cell studies.

## Figures and Tables

**Table 1:** Genes detected commonly in MDA-MB-231 and7 MCF-7 cells. Libraries that had at least one gene with 200 reads were qualified for mapping using RefSeq IDs. The genes displayed in the table were detected both in the MDA-MB-231 and MCF-7 cells.

RefSeq mRNA Accession	Gene Symbol	Gene Name
NM_001001521	UGP2	UDP-glucose pyrophosphorylase
NM_001002	RPLP0	ribosomal protein lateral stalk subunit P0
NM_001017963	HSP90AA1	heat shock protein 90 α family class A member 1
NM_001201483	ENO1	enolase 1
NM_001402	EEF1A1	eukaryotic translation elongation factor 1 α 1
NM_001699	AXL	AXL receptor tyrosine kinase
NM_002520	NPM1	nucleophosmin 1
NM_005324_mRNA	H3F3B	H3 histone, family 3B
NM_015932_mRNA	POMP	proteasome maturation protein
